# AGING: U.S. Lives: Longer but Sicker?

**DOI:** 10.1289/ehp.119-a118a

**Published:** 2011-03

**Authors:** Kris S. Freeman

**Affiliations:** **Kris S. Freeman** has written for *Encarta *encyclopedia, NIH, ABCNews.com, and the National Park Service. Her research on the credibility of online health information appeared in the June 2009 *IEEE Transactions on Professional Communication*

Contrary to society’s assumptions that good health will increase with each generation, Americans are living longer but enjoying fewer healthy years. “We do not appear to be moving to a world where we die without experiencing disease, functioning loss, and disability,” says Eileen M. Crimmins, a professor of gerontology at the University of Southern California (USC). According to an analysis^1^ by Crimmins and USC postdoctoral fellow Hiram Beltrán-Sánchez, between 1970 and 2005 the probability that a person aged 65 would live 20 more years doubled to 40%, primarily because of decreases in death from cardiovascular disease and cancer. However, average years spent living with morbidity^2^ also increased.

The analysis showed that a man who was 20 years old in 1998 could expect to live about 55 additional years, spending about 10 of those years with serious disease and 3.8 with limited mobility. In contrast, a man who was 20 years old in 2006 could expect to live longer still (56.1 additional years) but spend more time with disease (12.3 years) and lack of mobility (5.8 years). Women’s average life spans, although still about 5 years longer those of men, increased at a slower rate than men’s, while their years of morbidity increased at a higher rate.^1^

The United States lags behind many countries in increasing longevity. In 2010 the country ranked just 49th among world countries in life expectancy at birth.^3^ Crimmins and Beltrán-Sánchez note that recent increases in length of life in the United States have been caused more by improvements in treatments for disease than in disease prevention.^1^ For example, age-adjusted incidence of diagnosed diabetes^4^ doubled between 1980 and 2008 for both men and women.^5^ The authors state that only delaying the onset of disease through preventive care will lead to longer disease-free lives.^1^

However, “the reality is that prevention is not always easy and not always the best expenditure of money,” cautions David O. Meltzer, chief of hospital medicine at the University of Chicago. “Extensions of life expectancy lead to cost. We need to keep this in mind when evaluating cost-effectiveness. If we don’t, we may invest too much in things that extend the length of life and not enough on things that improve quality of life.” Avoiding difficult conversations about such matters, he adds, means “we will make mistakes, and we will be less healthy for it.”

Reducing exposure to pollutants may be one way to decrease morbidity and mortality. Crimmins and colleagues are analyzing the health impacts of exposure to air pollution, which Crimmins says is a clear risk factor for heart disease and cognitive loss. According to a study by the World Health Organization (WHO), exposure to coarse particulate air pollution above the WHO guideline of 20 μg/m3 annual mean caused an estimated 40,600 U.S. deaths in 2006.^6^

“At a time when we are all concerned about reducing climate change, it is remarkable that in many cities it is cheaper and more efficient to drive to work than to use public transport,” says Carlos Corvalan, a coauthor of the WHO analysis and a senior advisor on risk assessment and global environmental change for the Pan American Health Organization. “Addressing this single issue would go a long way in reducing emissions that cause air pollution and respiratory diseases in addition to reducing greenhouse gas emissions and climate change.”
